# Persimmon-derived tannin has antiviral effects and reduces the severity of infection and transmission of SARS-CoV-2 in a Syrian hamster model

**DOI:** 10.1038/s41598-021-03149-3

**Published:** 2021-12-08

**Authors:** Ryutaro Furukawa, Masahiro Kitabatake, Noriko Ouji-Sageshima, Yuki Suzuki, Akiyo Nakano, Yoko Matsumura, Ryuichi Nakano, Kei Kasahara, Kaoru Kubo, Shin-ichi Kayano, Hisakazu Yano, Toshihiro Ito

**Affiliations:** 1 Department of Immunology, Nara Medical University, Kashihara, Nara 634-8521 Japan; 2 Center for Infectious Diseases, Nara Medical University, Kashihara, Nara 634-8522 Japan; 3 Department of Microbiology and Infectious Diseases, Nara Medical University, Kashihara, Nara 634-8521 Japan; 4 Department of Health and Nutrition, Faculty of Health Science, Kio University, Kitakatsuragi-gun, Nara 635-0832 Japan; 5 MBT (Medicine-Based Town) Institute, Nara Medical University, Kashihara, Nara 634-8521 Japan; 6 Laboratory Animal Research Center, Nara Medical University, Kashihara, Nara 634-8521 Japan

**Keywords:** Immunology, Microbiology

## Abstract

Coronavirus disease 2019 (COVID-19) caused by severe acute respiratory syndrome coronavirus 2 (SARS-CoV-2) has rapidly spread across the world. Inactivating the virus in saliva and the oral cavity represents a reasonable approach to prevent human-to-human transmission because the virus is easily transmitted through oral routes by dispersed saliva. Persimmon-derived tannin is a condensed type of tannin that has strong antioxidant and antimicrobial activity. In this study, we investigated the antiviral effects of persimmon-derived tannin against SARS-CoV-2 in both *in vitro* and *in vivo* models. We found that persimmon-derived tannin suppressed SARS-CoV-2 titers measured by plaque assay *in vitro* in a dose- and time-dependent manner. We then created a Syrian hamster model by inoculating SARS-CoV-2 into hamsters’ mouths. Oral administration of persimmon-derived tannin dissolved in carboxymethyl cellulose before virus inoculation dramatically reduced the severity of pneumonia with lower virus titers compared with a control group inoculated with carboxymethyl cellulose alone. In addition, pre-administration of tannin to uninfected hamsters reduced hamster-to-hamster transmission of SARS-CoV-2 from a cohoused, infected donor cage mate. These data suggest that oral administration of persimmon-derived tannin may help reduce the severity of SARS-CoV-2 infection and transmission of the virus.

## Introduction

Since December 2019, we have been facing the global spread of newly-confirmed coronavirus disease 2019 (COVID-19), caused by severe acute respiratory syndrome coronavirus 2 (SARS-CoV-2)^1,2^. SARS-CoV-2 has spread rapidly and affected people worldwide since the World Health Organization (WHO) declared a pandemic on March 11, 2020^3^. There are several factors causing COVID-19 to spread so easily; one is that infected persons with no or mild symptoms can be infectious^4^, and another is that the virus is transmitted by dispersed saliva through oral routes^5^. Therefore, inactivating the virus in saliva and the oral cavity may be a reasonable approach to prevent human-to-human transmission.

The persimmon (*Diospyros kaki*) is an edible fruit mainly produced and consumed in East Asia, and astringent varieties contain large amounts of persimmon tannins, which are soluble polyphenols with strong astringency due to their strong adhesiveness to proteins^6^. The four types of catechin—epicatechin (EC), epicatechin gallate, epigallocatechin, and epigallocatechin gallate (EGCG)—are condensed into chains of 19–47 molecules long^7^. Because of its large molecular size, the persimmon tannin has strong antioxidant, immunomodulatory, and antimicrobial activities^6,8^. We have previously shown that dietary intake of the soluble fraction of persimmon-derived tannin attenuated *Mycobacterium avium* complex (MAC) infection in an *in vivo* mouse model^9^. Some reports have also shown that persimmon-derived tannin inactivates various viruses, including influenza virus and herpes simplex virus-1^10–12^.

Here, we evaluated the antiviral effects of persimmon-derived tannin against SARS-CoV-2 both *in vitro* and *in vivo*. We used the Syrian Golden hamster (*Mesocricetus auratus*), which has been reported to be a good preclinical model of COVID-19 in humans, studying disease progression, pathophysiology and transmission of SARS-CoV-2^13–16^.

## Results

### Persimmon-derived tannin inactivates SARS-CoV-2 in vitro in a dose- and time-dependent manner

**Figure 1:**
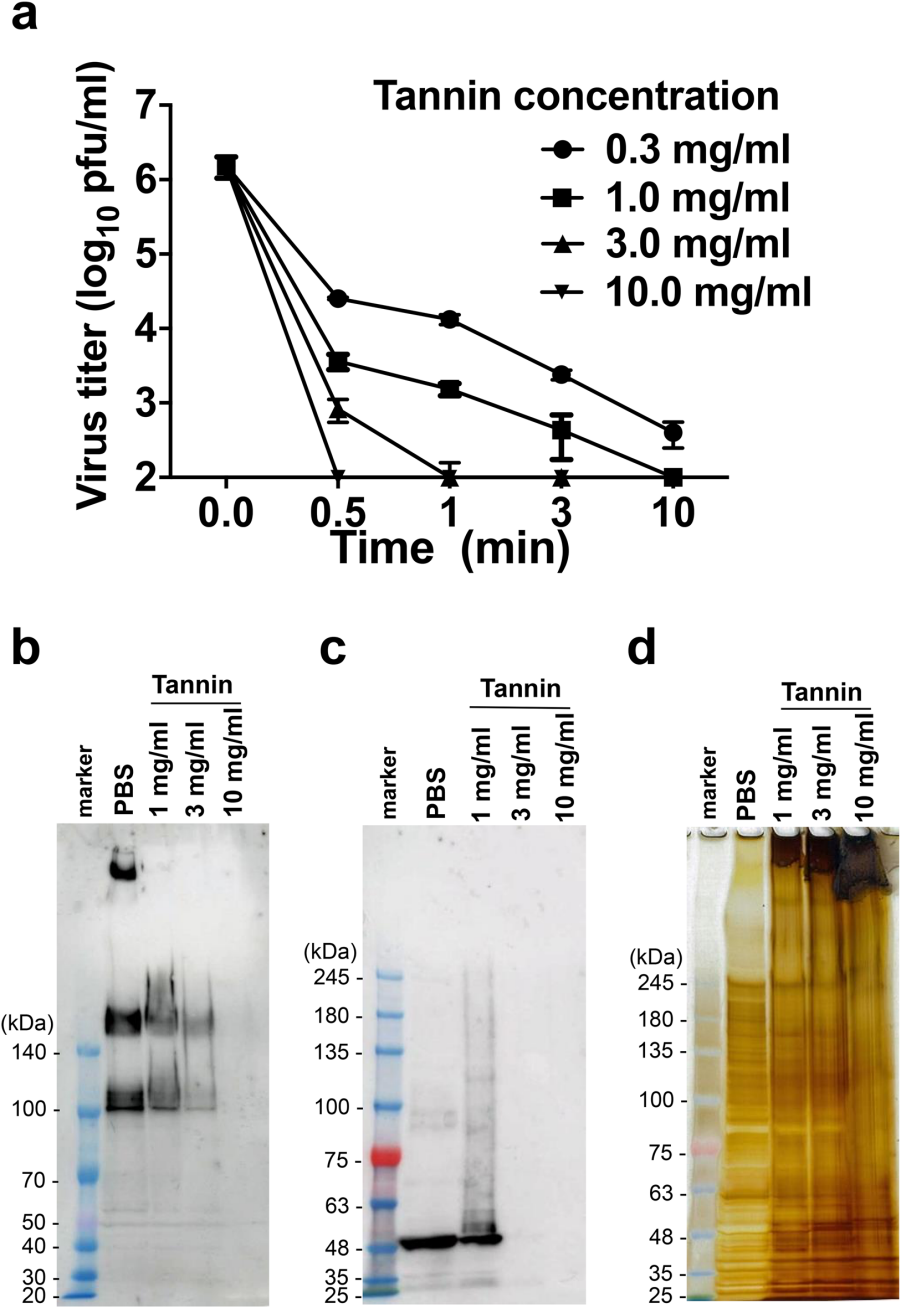
*In vitro* examination of persimmon-derived tannin against SARS-CoV-2. (**a**) Tannin powder was dissolved in distilled water and mixed with an amount of SARS-CoV-2 solution equal to 1.0 × 10^7^ plaque-forming units (pfu)/ml. After 30 s, 1 min, 3 min, and 10 min of reaction time, bovine serum albumin adjusted to 1% using PBS was added to block the tannin effect. The SARS-CoV-2 titers of each tannin-virus solution were measured using the plaque assay method. Data are shown as the mean ± SEM of three independent experiments. (**b–d**) SARS-CoV-2 (1.0 × 10^6^ pfu) was incubated with tannin (1, 3, and 10 mg/ml) or PBS for 3 min, then (**b**) SARS-CoV-2 spike and (**c**) nucleocapsid proteins were analyzed by western blotting and (**d**) sodium dodecyl sulfate polyacrylamide gel electrophoresis (SDS-PAGE) with silver staining. A major band at the top of the lane, and the major bands of approximately 180 kDa and 90 kDa are the trimeric, full-length, and cleaved spike proteins of SARS-CoV-2, respectively (**b**). A major band of approximately 55 kDa is the SARS-CoV-2 nucleocapsid protein (**c**). A major band at the top of the lane is an aggregate of tannin and viral proteins (**d**).

First, to investigate the antiviral activity of persimmon-derived tannin against SARS-CoV-2 in vitro, we examined the replicative ability of SARS-CoV-2 reacting with various concentrations of persimmon-derived tannin. We used EC and EGCG as negative and positive controls because EGCG, but not EC, has been reported to inhibit SARS-CoV-2 infection^17^. The results showed that persimmon-derived tannin had the ability to inactivate SARS-CoV-2 (JPN/TY/WK521/2020). This suppressive efficiency was dependent on both concentration and exposure time of persimmon-derived tannin (Fig. 1a), and stronger than that of EGCG (Fig. S1a). Importantly, we also found that persimmon-derived tannin was effective against the Alpha variant of SARS-CoV-2 (JPN/QHN001/2020) (Fig. S1b).

Next, we investigated the effect of persimmon-derived tannin on SARS-CoV-2 proteins by western blotting and sodium dodecyl sulfate polyacrylamide gel electrophoresis (SDS-PAGE) with silver staining (Fig. 1b–d). In the phosphate-buffered saline (PBS) control, three major bands were detected by rabbit anti-SARS-CoV-2 spike antibody, reflecting the trimeric, full-length (180 kDa) and cleaved (90 kDa) spike proteins of SARS-CoV-2, respectively (Fig. 1b), consistent with a previous report^18^. The SARS-CoV-2 nucleocapsid protein was also observed as a major band of 55 kDa (Fig. 1c). The density of bands of both spike and nucleocapsid proteins diminished depending on the dose of tannin, and completely disappeared by the incubation with 10 mg/ml of tannin (Fig. 1b,c). In contrast, silver staining detected bands at the tops of the lanes and these became denser depending on the tannin concentration (Fig. 1d). These results indicated that viral proteins formed large aggregates with persimmon-derived tannin, which could not react with antiviral antibodies due to the virus being covered by persimmon-derived tannin, as previously described^7^.

### Pre-administration of persimmon-derived tannin reduces virus titers and protects from pneumonia induced by SARS-CoV-2 infection in the hamster model

**Figure 2:**
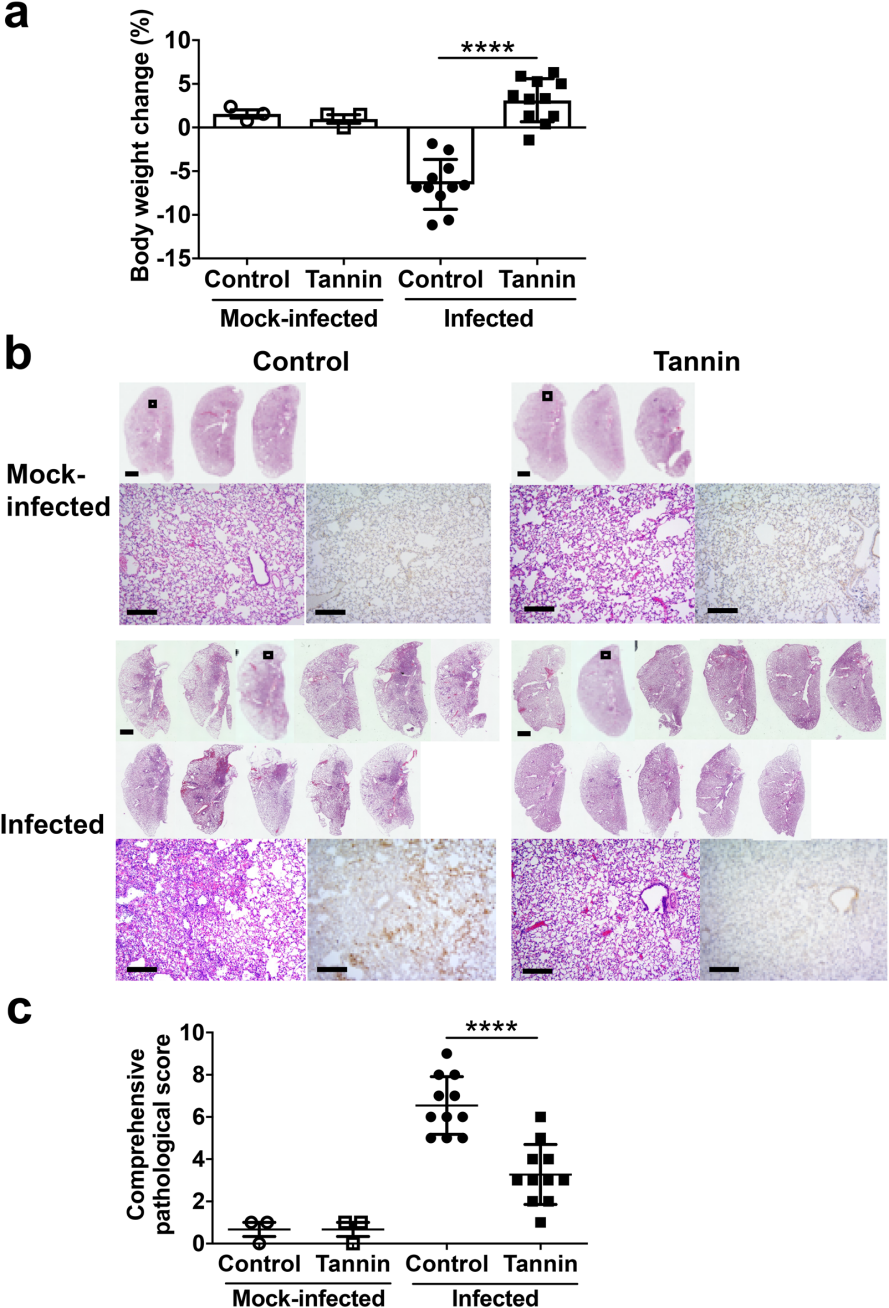
Effect of pre-administration of persimmon-derived tannin on body weight and histopathological changes in hamsters inoculated with SARS-CoV-2. Hamsters were inoculated with 1.0 × 10^5^ plaque-forming units of SARS-CoV-2 5 min after the administration of tannin or control solution to the tongue. They were euthanized 3 days after inoculation. (**a**) Changes in body weight at 3 days after inoculation (% weight change compared with the day of inoculation; Day 0). (**b**) Pathological findings in the left lungs of the hamsters. Views of the left lungs of hematoxylin and eosin (HE) staining are shown in the upper panels. Lower left panels show enlarged views of the area enclosed by the square in the upper panels; lower right panels show immunohistochemistry for SARS-CoV-2 antigen detection in the same area. Scale bars, 2 mm (upper panels) and 200 μm (lower left and lower right panels). (**c**) Comprehensive pathological score of left lung sections derived from HE staining. The scores were calculated based on the severity of pneumonia in the lungs of each hamster. Data are shown as the mean ± SEM of two independent experiments (mock-infected, control group, n = 3; mock-infected, tannin group, n = 3; infected, control group, n = 11; infected, tannin group, n = 11). ****p < 0.0001.

Next, we examined the *in vivo* antiviral and anti-inflammatory effect of persimmon-derived tannin using SARS-CoV-2-infected Syrian Golden hamsters. Anesthetized hamsters were administered tannin mixed with carboxymethyl cellulose (CMC) (tannin group) or CMC without tannin (control group) on the tongue. Five minutes after administration, hamsters were inoculated with 1.0 × 10^5^ plaque-forming units (pfu) of SARS-CoV-2 via the oral route. The control group hamsters lost body weight (− 6.51 ± 0.681%), whereas the tannin group hamsters increased their body weight on day 3 post infection (+ 3.12 ± 0.744%) (Fig. 2a). Histopathological analysis by hematoxylin and eosin (HE) staining revealed severe lung lesions with an increase in inflammatory cells and a large area of consolidation in the control group (Fig. 2b). Conversely, the tannin group hamsters showed much less lung inflammation. Comprehensive pathological scores of the tannin group were significantly lower than those of the control group (Fig. 2c). Furthermore, immunohistochemistry for the nucleocapsid protein of SARS-CoV-2 showed the tannin group hamsters had less viral antigen in the lungs compared with the control animals.

**Figure 3:**
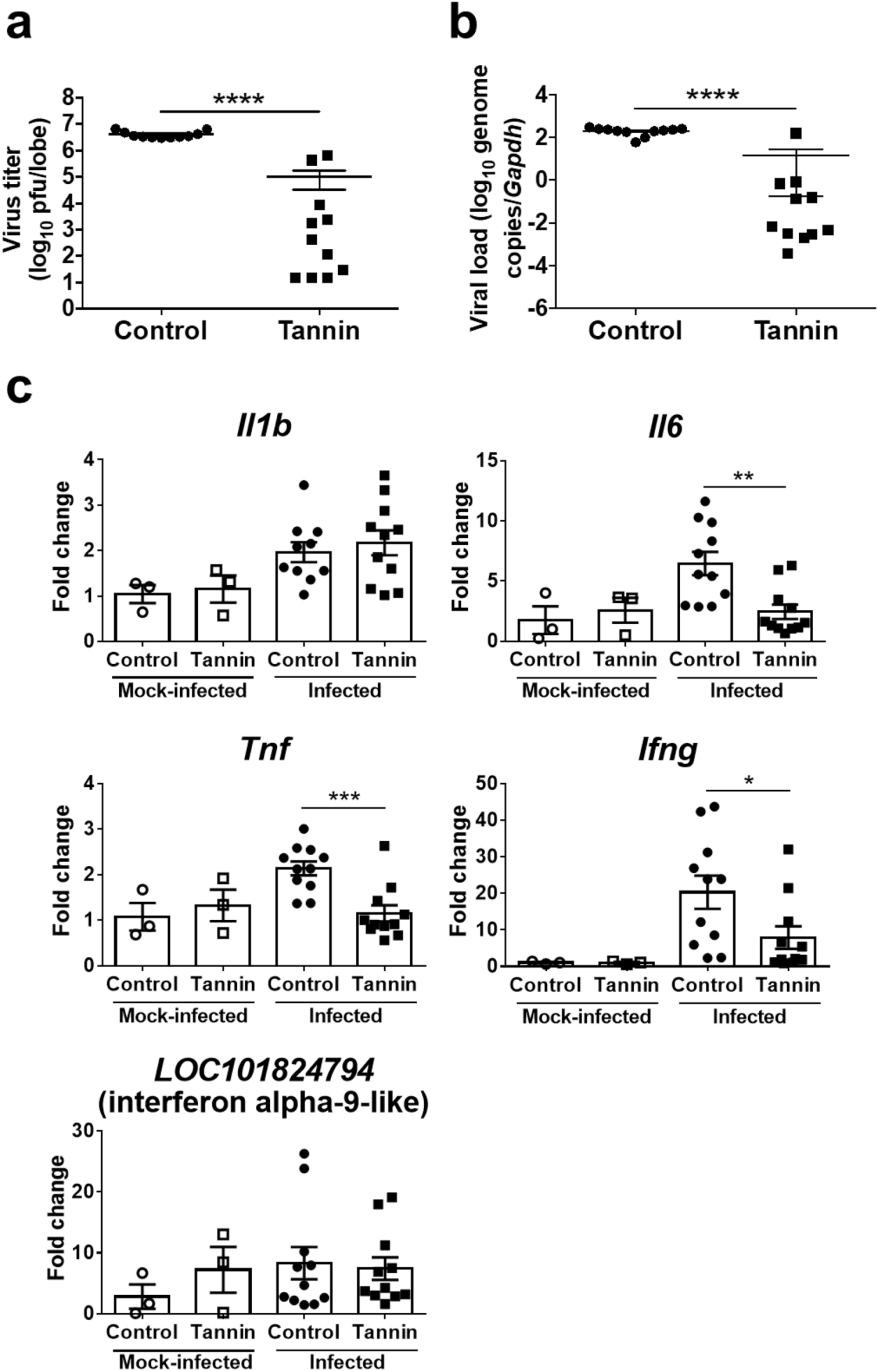
Effect of pre-administration of persimmon-derived tannin on virus titers, viral load, and inflammatory-related gene expression in the lungs of hamsters inoculated with SARS-CoV-2. (**a**) Virus titers were measured by the plaque assay method. The uppermost lobe of the right lung was used and plaque-forming units (pfu) per lobe were calculated. (**b**) Viral load was measured by quantitative PCR. (**c**) Expressions of *Il1b*, *Il6*, *Tnf*, *Ifng,* and *LOC101824794* (interferon alpha-9-like) were measured by quantitative PCR. RNA extracted from the right lung was used and normalized to *Gapdh* expression. Data are shown as the mean ± SEM of two independent experiments (mock-infected, control group, n = 3; mock-infected, tannin group, n = 3; infected, control group, n = 11; infected, tannin group, n = 11). *p < 0.05, **p < 0.01, ***p < 0.001, ****p < 0.0001.

Consistent with the histopathological data, plaque assay indicated significantly lower virus titers in the lungs of the tannin group compared with CMC controls on day 3 post infection (Fig. 3a). The SARS-CoV-2 viral load in the lungs detected by quantitative PCR (qPCR) was also lower in the tannin group hamsters than the control group (Fig. 3b). In addition, viral RNA extracted from the tongue tended to be lower in the tannin group, although the difference was not statistically significant (Fig. S2a).

Since high expression of inflammatory cytokines and interferon is associated with a strong inflammatory response in the lungs^19^, we further compared the gene expressions of *Il1b*, *Il6*, *Tnf*, *Ifng*, and *LOC101824794* (interferon alpha-9-like) in the lungs of both groups. The expressions of *Il6*, *Tnf*, and *Ifng* were significantly decreased in the tannin group, while the expression of *Il1b* and *LOC101824794* were comparable between the two groups (Fig. 3c).

### Pre-administration of persimmon-derived tannin to uninfected hamsters can reduce the transmission of SARS-CoV-2 from a cohoused donor cage mate

**Figure 4:**
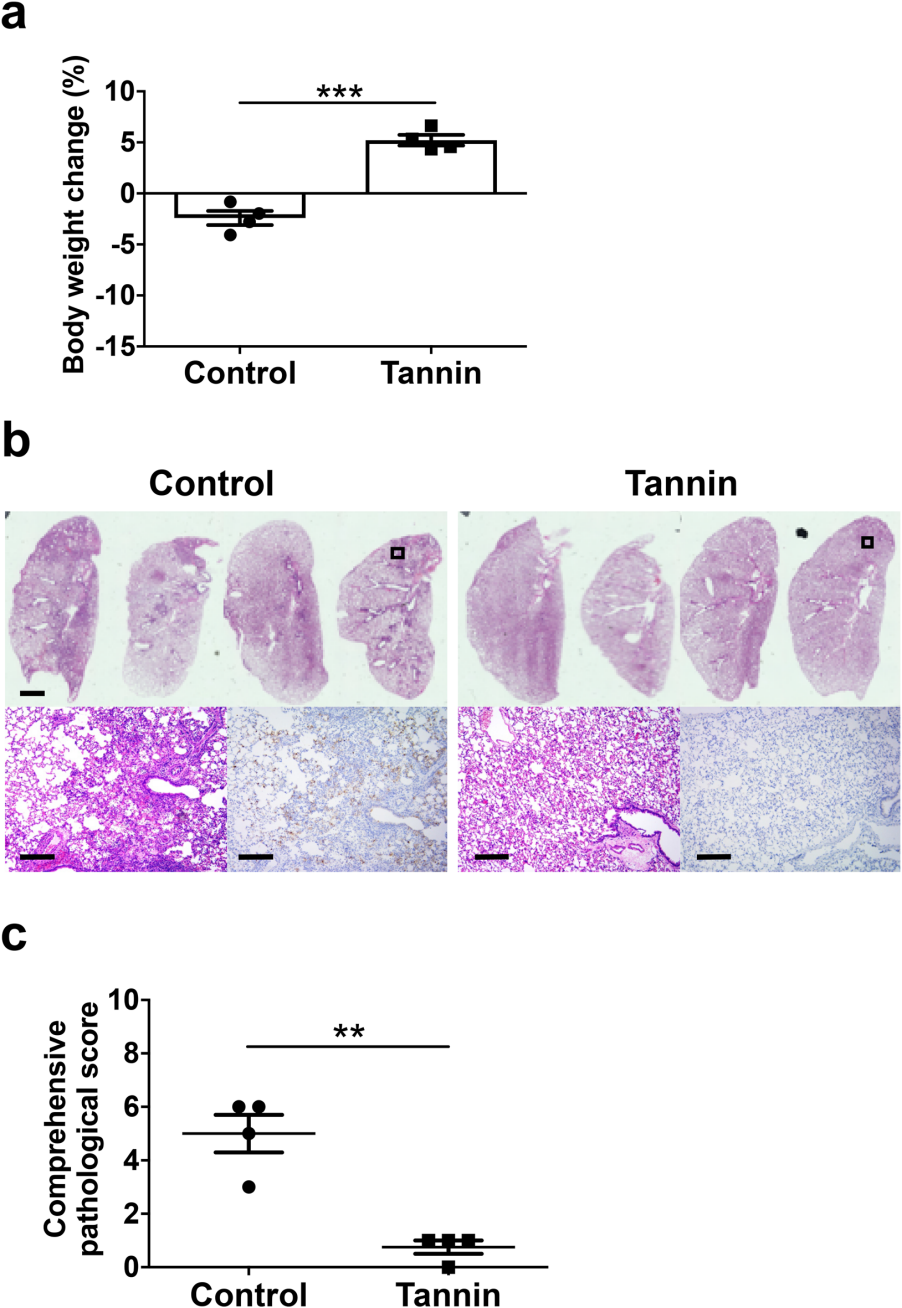
Effect of pre-administration of persimmon-derived tannin on body weight and histopathological changes in contact hamsters cohoused with a virus-inoculated donor. Uninfected hamsters administered with tannin or control solution to their tongue were cohoused with an inoculated donor hamster for 30 min and euthanized 7 days after contact. (**a**) Changes in body weight at 7 days after contact (% weight change compared with the day of contact). (**b**) Pathological findings in the left lungs of the contact hamsters. Views of the left lungs following hematoxylin and eosin (HE) staining are shown in the upper panels. Lower left panels show enlarged views of the area enclosed by the square in the upper panels; lower right panels show immunohistochemistry for SARS-CoV-2 antigen detection in the same area. Scale bars, 2 mm (upper panels) and 200 μm (lower left and lower right panels). (**c**) Comprehensive pathological score of left lung sections derived from HE staining. The scores were calculated based on the severity of pneumonia in the lungs of each hamster. Data are shown as the mean ± SEM (control group, n = 4; tannin group, n = 4). **p < 0.01, ***p < 0.001.

**Figure 5:**
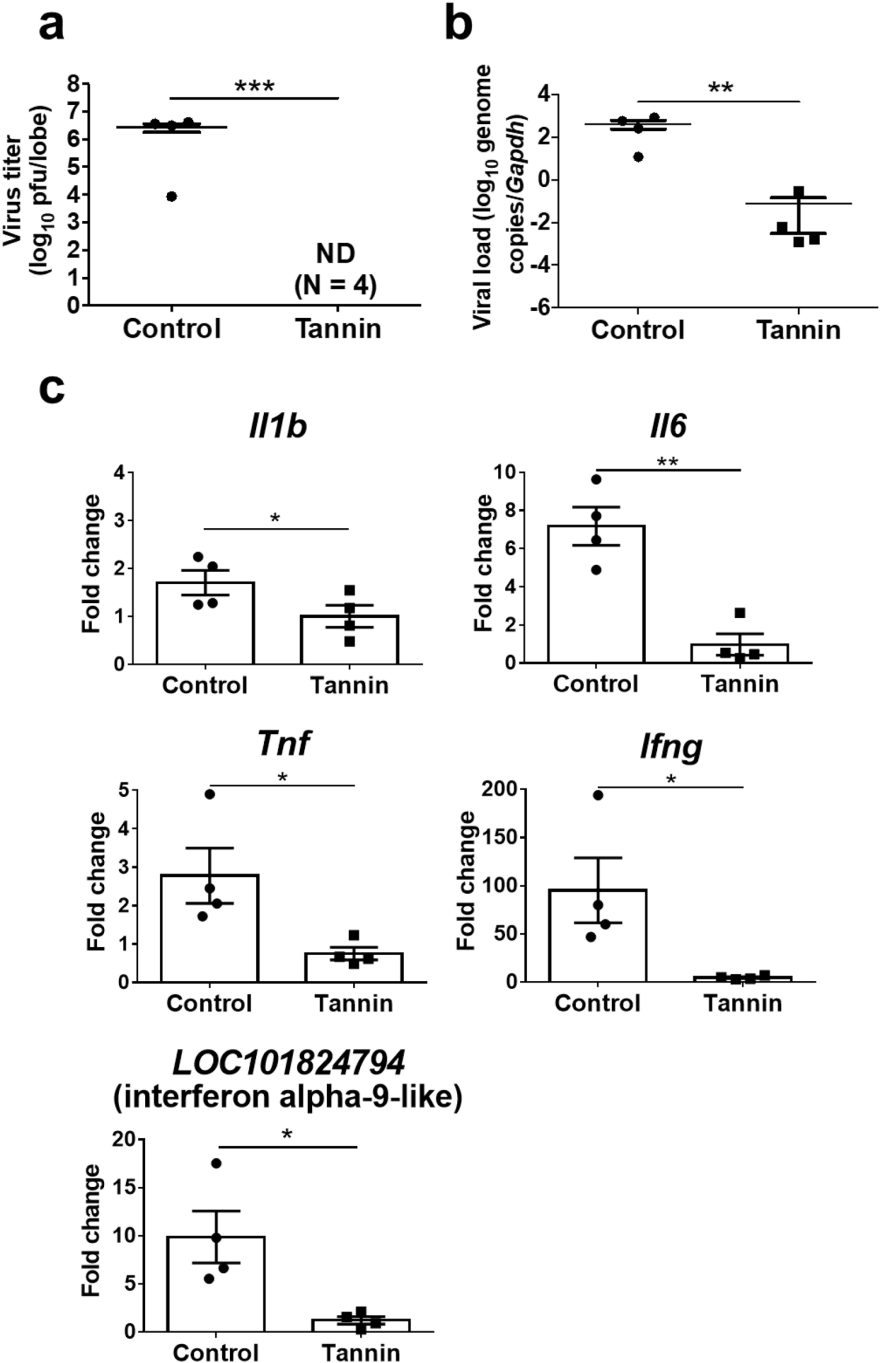
Effect of pre-administration of persimmon-derived tannin on virus titers, viral load, and inflammatory-related gene expression in the lung of contact hamsters cohoused with a virus-inoculated donor. (**a**) Virus titers were measured by plaque assay. The uppermost lobe of the right lung was used and the plaque-forming units (pfu) per lobe were calculated. (**b**) Viral load was measured by quantitative PCR. (**c**) Expressions of *Il1b*, *Il6*, *Tnf*, *Ifng*, and *LOC101824794* (interferon alpha-9-like) were measured by quantitative PCR. RNA extracted from the right lung was used and normalized to *Gapdh* expression. Data are shown as the mean ± SEM (control group; n = 4, tannin group, n = 4). ND, not detected. *p < 0.05, **p < 0.01, ***p < 0.001.

We next assessed the effects of tannin on transmission of SARS-CoV-2 in the hamster model. We orally inoculated one donor hamster with 1.0 × 10^5^ pfu of SARS-CoV-2. At 3 days after donor inoculation, uninfected hamsters were cohoused in the same cage with the donor hamster for 30 min, immediately after pre-administration of tannin or CMC on the tongue. Hamsters in the control group lost weight (− 2.41 ± 0.681%), whereas hamsters in the tannin group gained weight (+ 5.23 ± 0.520%) (Fig. 4a). Histological assessment revealed that hamsters in the tannin group developed less severe pneumonia with significantly lower comprehensive pathological scores (Fig. 4b,c), and showed lower detection of SARS-CoV-2 protein by immunohistochemistry compared with those in the control group (Fig. 4b). Consistent with the histopathological data, lung virus titers determined by plaque assay and lung viral load detected by qPCR were significantly lower in the tannin group compared with control animals (Fig. 5a,b). Viral load in the tongue was also lower in the tannin group hamsters compared with the control group (Fig. S2b). Moreover, gene expressions of inflammation-related cytokines *Il1b*, *Il6*, *Tnf*, *Ifng*, and *LOC101824794* in the lungs were significantly lower in the tannin group compared with those of the control group (Fig. 5c).

## Discussion

In our study, we first demonstrated that persimmon-derived tannin inactivated SARS-CoV-2 in vitro. The antiviral effect of tannin was dose- and time-dependent, and was observed not only against the nonvariant strain but also against the Alpha variant. The result was similar to a previous report of *in vitro* tannin effect against various viruses^12^. Western blotting and silver staining showed that persimmon-derived tannin induced aggregation of SARS-CoV-2. This aggregate-forming ability may be related to the antiviral effect against a variety of viruses, including variants of SARS-CoV-2. It is expected to be revealed more detailed mechanism of how persimmon-derived tannin can aggregate SARS-CoV-2 in future.

To examine the effects of tannins in vivo, we created a SARS-CoV-2 infection model by administering the virus into the oral cavity of anesthetized hamsters. According to a previous report^20^, hamsters in the oral inoculation group, which were given the virus through spontaneous self-swallowing without anesthesia, developed much less severe pneumonia than hamsters in the intranasal inoculation group with anesthesia. In our study, anesthetized hamsters aspirated the virus solution, and it might be the reason why they developed severe pneumonia, as in previous studies^16,20–22^ with intranasal administration. The severity of SARS-CoV-2-related lung lesions depends on how high virus titers reach the lower respiratory tract in the hamster model. Therefore, the effect of tannins *in vivo* demonstrated herein could be mainly due to the reduction of active virus in the oral cavity. Viral loads in the tongue of infected hamsters, which tended to be lower in the tannin group, were remarkably lower than that in the lung, partly because the virus in the oral cavity had already been reduced by the day of the dissection. To further demonstrate the effect of tannins, it may be necessary to examine viral loads in the oral cavity immediately after tannin administration. Also, it would be interesting to investigate whether persimmon-derived tannins have the ability to rescue the lung associated pathologies and mitigate the elevated pro-inflammatory cytokine response. Further experiments are needed to show the effect of tannins administered after infection.

We also found that oral administration of persimmon-derived tannins to uninfected hamsters prevented virus transmission from a donor cage mate. The effects shown in our contact experiments seem to be more consistent than those shown in the direct infection models. One possible reason for this is that hamsters in the contact experiments were exposed to lower titers of SARS-CoV-2, and most of the viruses were inactivated by the oral tannin treatment. However, in the direct infection models, the higher virus titers administered were not completely inactivated by tannins, which resulted in inconsistent severity of lung disease in directly inoculated hamsters.

Our contact experiment simulated a group of people in a crowded situation. The results indicate that tannins in the mouth may prevent infections in a place crowded with many people. While we administered tannins to uninfected recipient hamsters, experimental inoculation of tannins to SARS-CoV-2-infected donor hamsters would be useful to further evaluate the effect of tannin on SARS-CoV-2 transmission. In addition, the duration of tannin effect in the oral cavity is important. Tannins are expected to remain present for a relatively long time because they easily adhere to cells and viruses, but it is unclear how long tannins can actually continue to inactivate viruses in the mouth. Further study is needed to show when and how tannins should be administered to prevent transmission of SARS-CoV-2.

In summary, we clearly demonstrate that persimmon-derived tannin ameliorates the pathogenesis of COVID-19 by suppression of SARS-CoV-2 titers and inflammatory responses in the lungs, not only by direct inactivation of SARS-CoV-2 but also by reducing its transmission. Persimmon-derived tannin is a highly safe product extracted from a natural plant and may be a valuable candidate for COVID-19 protection and medical therapy.

## Materials and methods

### Tannin preparation

The procedure for persimmon-derived tannin extraction has been described previously^8,9^. Immature persimmon fruits (Ebenaceae *Diospyros kaki* Thunb., cv. ‘Hiratane-nashi’ and ‘Tone-wase’) harvested in Nara, Japan, in 2011 were used for extraction of tannin. ‘Hiratane-nashi’ was designated as a protected species No.24, Niigata Prefecture, Japan in 1962. ‘Tone-wase’, mutated variety of ‘Hiratane-nashi’, was registered for ‘The Plant Variety Protection No.28’, Ministry of Agriculture, Forestry and Fisheries, Japan in 1980. Both cultivars are deposited in Persimmon Cultivar Genetic Resource by Nara Prefecture Agricultural Research and Development Center, Japan (‘Hiratane-nashi’ No. 135, ‘Tone-wase’ No. 118). In this study, the species of persimmon were identified by Dr. Sadahiro Hamasaki (Nara Prefecture Agricultural Research and Development Center, Japan). Immature persimmon fruits were treated with 0.2% ethanol for 5 days for insolubilization of tannin. The ethanol-treated persimmons were crushed into small pieces and soaked in water for 2 days. The supernatant contained soluble components such as sugars, and the residue contained insoluble tannin. After the supernatant was discarded, water was added to the residue. Insoluble tannin was changed into soluble tannin by heating at 120 °C for 30 min, then extracted in water. The extracted soluble tannin was then filtered, evaporated in vacuo, and dried at 160 °C. A batch of soluble tannin powder contained 75.5% condensed tannin, measured as epigallocatechin gallate equivalent based on the Folin–Ciocalteau method. Soluble persimmon tannin powder was provided by Ishii Bussan Inc. (Nara, Japan) and stored at − 20 °C until use as previously described^10^. The cultivation and harvest of persimmon fruits by Ishii Bussan Inc. was permitted by The Agricultural Committee in Tenri City, Nara, Japan. All procedures were performed in accordance with World Health Organization (WHO) guidelines on good agricultural and collection practices (GACP) for medicinal plants. EC and EGCG were purchased from Sigma-Aldrich (Taufkirchen, Germany) under product numbers E4018 and E4143, respectively.

### Virus preparation and biosafety

SARS-CoV-2 nonvariant nCoV-19/JPN/TY/WK521/2020 and Alpha variant nCoV-19/JPN/QHN001/2020 were isolated and provided by the National Institute of Infectious Diseases, Japan. Virus culture was performed using VeroE6/TMPRSS2 cells (JCRB Cell Bank in Japan, JCRB1819). The virus culture fluid was subjected to two cycles of freezing and thawing, and centrifuged at 10,000 × *g* for 15 min at 4 °C. The supernatant was then ultrafiltered using Amicon Ultra-15 (Merck, Darmsthadt, Germany) and washed with PBS for preparing virus extracts^23^. The virus stocks were titrated to determine pfu in VeroE6/TMPRSS2 cells using the plaque assay method as described below. Viral load was measured by qPCR for viral antigens as described below. All experiments using SARS-CoV-2 were performed at the biosafety level (BSL) 3 experimental facility at Nara Medical University, Japan.

### *In vitro* SARS-CoV-2 infection

Tannin powder was diluted with distilled water. The virus stock was diluted to 1.0 × 10^7^ pfu/ml in PBS. EC, EGCG, or persimmon-derived tannin and an equal amount of virus solution were mixed to inactivate the virus in vitro. After varying periods of time for inactivation of the virus, bovine serum albumin (BSA) (Fujifilm Wako Pure Chemical Corporation, Osaka, Japan) that was adjusted to 1% using PBS was added to block the tannin effect. Each tannin-virus solution was then serially diluted tenfold using PBS and administered to VeroE6/TMPRSS2 cells. Virus titers were measured using the plaque assay method as described below.

Viral proteins treated with persimmon-derived tannin were analyzed by western blotting and SDS-PAGE with silver staining. SARS-CoV-2 solution containing 1.0 × 10^6^ pfu was incubated with an equal volume of tannin solution (1, 3, and 10 mg/ml) or PBS control for 3 min. The samples were mixed with 2 × SDS buffer and denatured at 50 °C for 10 min, and then kept at − 80 °C until use. The prepared samples were analyzed by gel electrophoresis using a 5–12% gradient gel (SuperSep™ ACE; Fujifilm Wako Pure Chemical Corporation) and western blotting. To detect SARS-CoV-2 spike and nucleocapsid proteins by western blotting analysis, a rabbit antiSARS-CoV-2 spike polyclonal antibody (Cat No. 40150-T62-COV2; Sino biological Inc., Beijing, China) at 1/1,000 dilution and a rabbit anti-SARS-CoV-2 nucleocapsid polyclonal antibody (Cat No. NB100-56576; Novus Biologicals, Littleton, CO) at 1 µg/ml were used, respectively. These antibodies were incubated with the membrane overnight at 4 °C and then, washed with PBS containing 0.05% Tween-20 (Fujifilm Wako Pure Chemical Corporation). To visualize these reactions, anti-rabbit IgG-HRP (Santa Cruz Biotechnology, Dallas, TX) and chemiluminescence (Western Lightning Ultra; PerkinElmer, Waltham, MA) were used. Silver staining was performed using 2D-Silver Stain Reagent II (Cosmo Bio Co., Tokyo, Japan) in accordance with the manufacturer’s instructions.

### Animals

Six-week-old, male Syrian Golden hamsters (*Mesocricetus auratus*) were purchased from Japan SLC (Hamamatsu, Shizuoka, Japan) and kept in a specific pathogen-free environment at the Animal Research Center of Nara Medical University. The animal experimental protocols were approved by The Animal Care and Use Committee at Nara Medical University (Approval Number, 12922). All procedures were performed in accordance with the Policy on the Care and Use of Laboratory Animals, Nara Medical University and Animal Research: Reporting of In Vivo Experiments (ARRIVE) guidelines.

### Direct infection model

Before virus inoculation, they were weighed and anesthetized with an intraperitoneal injection of pentobarbital. In the tannin group, 20 mg of tannin powder mixed with 100 μl of 0.5% CMC (Nacalai Tesque, Kyoto, Japan) was applied on the tongue. Five minutes later, 50 μl of virus solution diluted with saline (including 1.0 × 10^5^ pfu of SARS-CoV-2) was administered into the mouth. In the control group, 0.5% CMC without tannin powder was applied to the tongue and 50 μl of virus solution was administered. At 3 days after inoculation, the SARS-CoV-2-challenged hamsters were weighed and euthanized. Lungs and the tongue were sampled for histopathological examination, virus titration, and qPCR.

### Hamster-to-hamster infection model

We inoculated one donor hamster with 1.0 × 10^5^ pfu of SARS-CoV-2 via the oral route as described above. At 3 days after donor inoculation, the contact experiment was performed. Uninfected hamsters were distributed into two groups. In the tannin group, 20 mg of tannin powder mixed with 100 μl of 0.5% CMC was applied to the tongue. For the control group, 100 μl of 0.5% CMC without tannin powder was applied to the tongue. Hamsters were anesthetized by isoflurane inhalation prior to administration of tannin or control solution. The uninfected hamsters were transferred to the same cage and cohoused with the donor hamster immediately after the pre-administration of tannin or control solutions. Both groups of four hamsters were cohoused with the donor for 30 min. Seven days later, they were weighed and euthanized. Lungs and the tongue were sampled for histopathological examination, virus titration, and qPCR.

### Histological analysis

The left lungs of hamsters were fixed with 4% paraformaldehyde. After embedding in paraffin, sections were sliced to a thickness of 1–2 μm and stained with HE (Sakura Finetek Japan Co., Tokyo, Japan). The pathological score assessment used in this study was derived from a recent study^24^. (a) Alveolar septum thickening and consolidation, (b) hemorrhage, exudation, pulmonary edema, and mucous, and (c) recruitment and infiltration of inflammatory cells were pathologically examined. For each category, scores were determined according to the severity of the disease as follows: 0, no observable pathological change; 1, mild pathological change; 2, moderate pathological change; 3, severe pathological change; 4, very severe pathological change. The comprehensive pathological score was the sum of the scores for each of the three categories. Blinded histological assessment was performed by a trained pathologist. SARS-CoV-2 viral antigens were detected by immunohistochemistry. Briefly, sections were deparaffinized in xylene and rehydrated in a sequence of descending concentrations of ethanol. Sections were subjected to heat-induced epitope retrieval with HistoVT One (Nacalai Tesque) at 121 °C for 15 min. Endogenous peroxidase reactivity was blocked with 3% H_2_O_2_ for 10 min, and then sections were incubated with a rabbit anti-SARS-CoV-2 nucleocapsid polyclonal antibody (2 µg/ml, NB100-56576; Novus Biologicals) overnight at 4 °C. After washing, sections were treated with peroxidase-labeled anti-rabbit IgG (Histofine Simplestain rat-MAX-PO (MULTI)) (Nichirei Bioscience, Tokyo, Japan) in accordance with the manufacturer’s instructions. Histochemical reactions were developed using 3, 3-diaminobenzidine (DAB) (DAB Substrate Kit, Cat No. 425011; Nichirei Bioscience) as the chromogenic substrate for peroxidase. Finally, sections were counterstained with hematoxylin, dehydrated, and mounted.

### Determination of virus titers by plaque assay

The titers of infectious SARS-CoV-2 were measured by plaque assay. VeroE6/TMPRSS2 cells were grown to confluence on 12-well plates. Virus solution, virus solution mixed with tannin powder, or a solution of homogenized hamster lungs was serially diluted tenfold with PBS and administered to the cells. The uppermost of the four lobes of each hamster’s right lung was homogenized using the gentleMACS Dissociator (Miltenyi Biotec, Bergisch Gladbach, Germany). After virus inoculation, the cell monolayer was covered with agarose (Nippon Gene, Tokyo, Japan) overlay and incubated for 48 h at 37 °C with 5% CO_2_. After the agarose overlay was removed, the cells were fixed with 10% formalin, and stained with crystal violet. The number of visible plaques was counted, and the pfu of each solution was determined. Active virus titers are expressed as pfu/ml of the solution, or pfu/lobe of the hamster lung.

### qPCR

A part of the removed lungs and the tongue were immersed in 0.5 ml RNAlater^®^ Stabilization Solution (Thermo Fisher Scientific, Waltham, MA) overnight at 4 °C, and stored at − 80 °C until RNA extraction. The samples were homogenized and RNA was extracted from the tissues using NucleoSpin^®^ RNA in accordance with the manufacturer’s instructions (Macherey–Nagel, Düren, Germany). Total RNA was reverse transcribed to cDNA using a High-Capacity cDNA Reverse Transcription Kit in accordance with the manufacturer’s instructions (Thermo Fisher Scientific). qPCR was performed using a StepOnePlus Real-Time PCR System (Thermo Fisher Scientific). *Gapdh* expression was analyzed by TaqMan assay Cg04424038. qPCR for the expression of cytokine genes was performed using Fast SYBR Green Master Mix (Thermo Fisher Scientific) with specific primer sets (Table 1). The SARS-CoV-2 viral load was determined by detecting the nucleocapsid gene of the virus by qPCR. Referring to the manual prepared by the National Institute of Infectious Diseases, Japan^25^, we performed qPCR using forward primer (5′-AAATTTTGGGGACCAGGAAC-3′), reverse primer (5′-TGGCAGCTGTGTAGGTCAAC-3′), and the TaqMan probe (FAM-ATGTCGCGCATTGGCATGGA-BHQ). Gene expression was analyzed by the relative ΔΔCT method and normalized to *Gapdh* expression.

**Table 1:** Sequences of primers for quantitative PCR detection of cytokines in hamsters.

Gene name	Forward primer (5′ to 3′)	Reverse primer (5′ to 3′)
*Il1b*	GTGGACAACAAAGCTCGTGG	AGCCCGTCAACCTCAAAGAA
*Il6*	TGTCTTCTTGGGACTGCTGC	CCAAACCTCCGACTTGTTGA
*Tnf*	CACCCACCGTCAAGGATTCA	TTGGCTGGGCAATGAAGAGT
*Ifng*	TGCATCTTGGCTTTGTTGCTC	TCCCCTCCATTCACGACATC
*LOC101824794* (interferon alpha-9-like)	AGACTGGGAGTTGCCTGTGA	GAGGAATCCAGGGCTTTCCAG

### Statistical analysis

Data are presented as the mean ± SEM and are representative of at least two independent experiments. Statistical analyses of weight change, virus titers, and in vitro gene expression were performed by Student’s t-test using GraphPad Prism 6 (GraphPad Software, San Diego, CA). P values of < 0.05 were considered statistically significant.

## Supplementary Information

Supplementary Figures.
