# Supplementary material for: Persimmon-derived tannin has antiviral effects and reduces the severity of infection and transmission of SARS-CoV-2 in a Syrian hamster model
Source: Sci Rep. 2021 Dec 8;11:23695. doi: 10.1038/s41598-021-03149-3 (PMC8654961; doi:10.1038/s41598-021-03149-3)
Supplement: Supplementary file 1 — Supplementary Figures. [file 41598_2021_3149_MOESM1_ESM.pdf]

Supplementary information

Persimmon-derived tannin has antiviral effects and reduces the severity of infection and transmission of SARS-CoV-2 in a Syrian hamster model

Ryutaro Furukawa, Masahiro Kitabatake, Noriko Ouji-Sageshima, Yuki Suzuki, Akiyo Nakano, Yoko Matsumura, Ryuichi Nakano, Kei Kasahara, Kaoru Kubo, Shin-ichi Kayano, Hisakazu Yano, and Toshihiro Ito

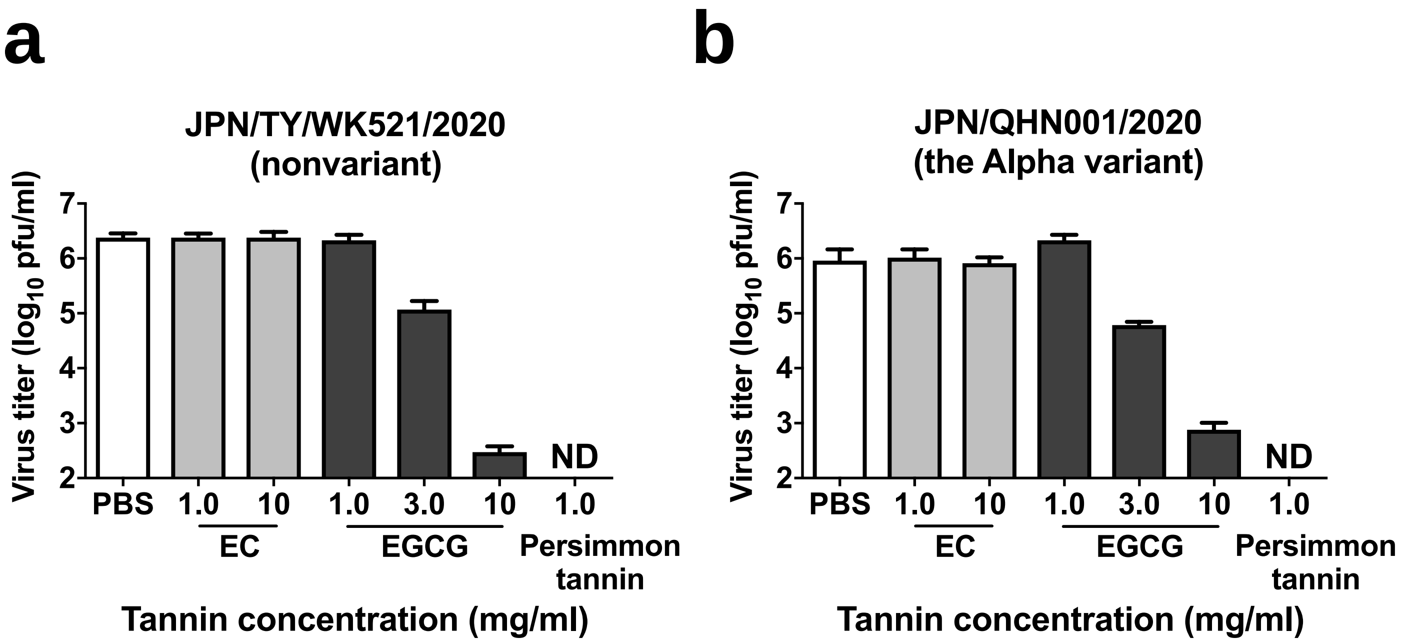

Supplementary Figure S1. *In vitro* examination of different types of tannin against SARS-CoV-2 strain of (**a**) JPN/TY/WK521/2020 (nonvariant) and (**b**) JPN/QHN001/2020 (the Alpha variant). Epicatechin (EC), epigallocatechin gallate (EGCG), and persimmon-derived tannin powder was dissolved in distilled water and mixed with an amount of SARS-CoV-2 solution equal to 1×10^7 plaque-forming units (pfu)/ml. After 10 minutes of reaction time, bovine serum albumin adjusted to 1% using PBS was added to block the tannin effect. The SARS-CoV-2 titers of each tannin-virus solution were measured using the plaque assay method. Data are shown as the mean ± SEM of three independent experiments. EC, epicatechin; EGCG, epigallocatechin gallate; ND, not detected.

Supplementary Figure S1

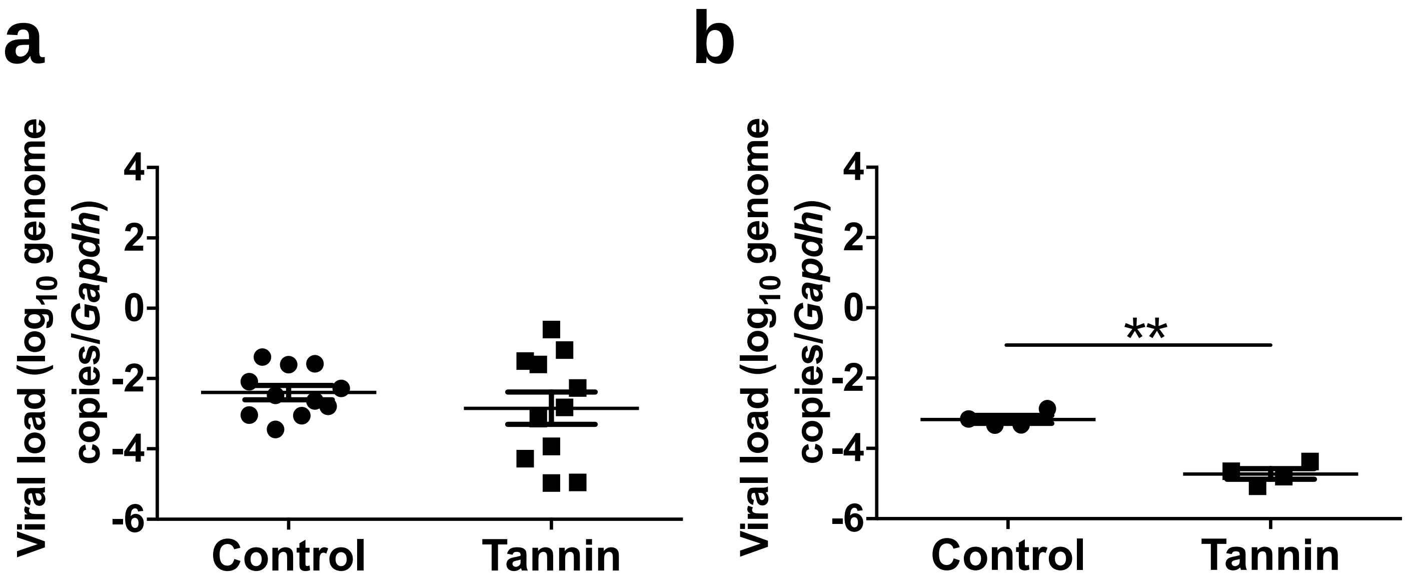

Supplementary Figure S2. Viral load in the tongue of (**a**) hamsters directly inoculated with SARS-CoV-2 (control group, n = 11; tannin group, n = 11) and (**b**) hamsters cohoused with a virus-inoculated donor (control group, n = 4; tannin group, n = 4). Viral load was measured by quantitative PCR. RNA extracted from the tongue was used and normalized to *Gapdh* expression. Data are shown as the mean ± SEM. $^{**}p < 0.01$.

Supplementary Figure S2
